# Current advance care planning practice in the Australian community: an online survey of home care package case managers and service managers

**DOI:** 10.1186/s12904-015-0018-y

**Published:** 2015-04-23

**Authors:** Marcus Sellars, Karen M Detering, William Silvester

**Affiliations:** Respecting Patient Choices, Austin Health, Melbourne, Australia; Respecting Patient Choices Program, Austin Hospital, PO Box 5555, Heidelberg, Melbourne, VIC 3084 Australia

**Keywords:** Advance care planning, Advance directives, Community health services, Home care services

## Abstract

**Background:**

Advance care planning (ACP) is the process of planning for future healthcare that is facilitated by a trained healthcare professional, whereby a person’s values, beliefs and treatment preferences are made known to guide clinical decision-making at a future time when they cannot communicate their decisions. Despite the potential benefits of ACP for community aged care clients the availability of ACP is unknown, but likely to be low. In Australia many of these clients receive services through Home Care Package (HCP) programs. This study aimed to explore current attitudes, knowledge and practice of advance care planning among HCP service managers and case managers.

**Methods:**

An invitation to take part in a cross-sectional online survey was distributed by email to all HCP services across Australia in November 2012. Descriptive analyses were used to examine overall patterns of responses to each survey item in the full sample.

**Results:**

120 (response rate 25%) service managers and 178 (response rate 18%) case managers completed the survey. Only 34% of services had written ACP policies and procedures in place and 48% of case managers had previously completed any ACP training. In addition, although most case managers (70%) had initiated an ACP discussion in the past 12 months and viewed ACP as part of their role, the majority of the conversations (80%) did not result in documentation of the client’s wishes and most (85%) of the case managers who responded did not believe ACP was done well within their service.

**Conclusions:**

This survey shows low organisational ACP systems and support for case managers and a lack of a normative approach to ACP across Australian HCP services. As HCPs become more prevalent it is essential that a model of ACP is developed and evaluated in this setting, so that clients have the opportunity to discuss and document their future healthcare wishes if they choose to.

**Electronic supplementary material:**

The online version of this article (doi:10.1186/s12904-015-0018-y) contains supplementary material, which is available to authorized users.

## Background

Advance care planning (ACP) is the process of planning for future healthcare that is facilitated by a trained healthcare professional, whereby a person’s values, beliefs and treatment preferences are made known so they can guide clinical decision making at a future time when that person cannot make or communicate their decisions due to lack of capacity. Ideally ACP involves a coordinated communication process between a patient, their family and healthcare providers. A desirable outcome of the ACP conversation is the completion of a written Advance Care Directive (ACD) that documents the person’s wishes and/or the appointment of a substitute decision-maker [1,2]. ACP has been shown to improve the likelihood that doctors and family members will know and comply with the patients’ wishes [3-6], lead to less aggressive care at the end of life [6-8], improve patient and family satisfaction with care, as well as reduced stress, anxiety and depression in surviving relatives [4-6,9].

Australia is constitutionally a federation with each state and territory having their own legislation regarding ACP. In the past 10 years, ACP has gained prominence in Australia because of emergence of legislation supporting ACP in each state and territory [1], through government support and funding and through promotion by health professionals and organisations [2]. Most ACP services within Australia at present are provided within hospitals, rehabilitation services and Residential Aged Care Facilities (RACFs).

In Australia since the early 1990’s, home care services have been available as an alternative to living in a RACF for older persons with complex care issues who would prefer to live in their own home. In Australia this is referred to as a Home Care Package (HCP). HCP services are funded by the Australian Government to facilitate the provision of personal support and clinical care services to elderly clients (aged >65 years), so that they can remain at home for as long as possible. Service providers tender to government for the right to provide HCPs. The service manager, who supervises the case managers, ensures that HCPs are delivered to clients in accordance with government guidelines. HCP clients are assigned a case manager who, in collaboration with the client and family, coordinates and reviews the care services that clients receive under the funding allocated to the HCP [10].

The availability of HCPs is set to increase where it is predicted 80% of aged care services will be delivered in the community by 2050 and that HCP packages will increase from 60,000 in 2012 to 100,000 by 2016–2017. The scope of services provided by HCPs has also expanded under the 2013 Consumer Directed Care reforms, including giving clients greater control as they are now able to choose the types of care that they receive [10].

Persons receiving HCPs, by definition are frail or have multiple medical problems with complex care needs. Such people are at risk of a deterioration in their health status. Generally elderly people in their own homes are less likely to have significant dementia than people in RACFs and, therefore, are able to participate in ACP. They will often be seen by health professionals in their own home [11], which may be a more comfortable environment for these conversations than the RACF or hospital setting [12], sites where ACP conversations are currently initiated. Research suggests that elderly people believe ACP is important and they want the opportunity to complete ACP [13]. Recipients of HCPs will typically have less dependency and health deterioration compared to persons in RACFs [14] or inpatient settings. Therefore offering ACP within HCPs may lead to earlier conversations and documentation of wishes, before an acute episode that may render the elderly person too unwell to express their wishes.

Despite the potential benefits of completing ACP discussions with HCP clients, the literature has not examined whether ACP can be successfully implemented in Australian HCP programs, or how this may be achieved. Past limited international research has shown that in general the uptake of ACP within community care is low [15-18] and that case managers vary in their ACP practice, knowledge and attitudes, based on their own personal experiences and qualifications [17,18]. Despite this, international surveys indicate that the majority of case managers [16,17,19] and clients [9] believe ACP is a worthwhile and valuable activity.

The aim of the study was to conduct a national online survey of those organisations that provide HCPs and assess current knowledge, attitudes, and practice in relation to ACP at both the organisational and case manager level. This information will be used to identify and address gaps relating to current ACP practices in HCPs and to explore the feasibility of case managers completing ACP conversations with clients receiving HCPs.

## Methods

### Study design and participants

All HCP services across Australia were identified through a contact list provided by the Commonwealth Department of Health and invited them to complete an online survey. The survey tool was accessible from November 2012 until January 2013.

The survey was distributed in two forms, one for case managers and one for service managers.

The email to the services invited them to nominate two case managers and one service manager to complete the online surveys, which were anonymous. Participation in the survey was voluntary and completion of the survey implied consent. Ethics approval for the study was obtained from the Austin Health Human Research Ethics Committee (Approval number H2012/04810).

### The survey tools

The surveys were designed to measure the current policies and procedures that are available in HCP organisations and the perspectives, attitudes and current practices of HCP case managers regarding ACP (Additional file 1). The surveys were developed with reference to existing US literature [5,16,17] and our past research examining ACP prevalence and practices in Residential Aged Care Facilities [20].

The HCP organisation survey, completed by service managers, comprised 11 questions focused on HCP service characteristics, 6 questions on ACP policies and procedures to assess their availability and dissemination to staff and 17 questions focused on organisational ACP practices.

The HCP Case Manager survey comprised 14 questions about the HCP service characteristics, 6 questions on ACP policy and procedures, 10 questions on past experience with ACP, 4 questions on attitudes and beliefs towards ACP and 12 demographic questions.

### Analysis

The data were analysed using descriptive statistics. Descriptive analyses were undertaken using SPSS for Windows. Frequencies and corresponding percentages are reported for categorical data. In addition, Chi square analysis was completed to examine the relationship between having a nurse training background (versus no nurse training background) and confidence levels on 8 separate ACP domains.

## Results

### Demographics and professional information

The invitation was sent to 481 valid email addresses and, in response 120 service managers and 178 case managers completed the survey. This would suggest a response rate of 25% for service managers and 18% for case managers^1^. The majority of case managers were aged 40 years or older, 90% (n = 160) were female and 42% (n = 75) were from a nursing training background, although there was a wide variation in their training classifications (Table 1). All Australian states and the territories were represented apart from the Australian Capital territory (1.6% of Australian population) [21]. All funding models of service providers were represented (Table 2).

**Table 1:** **Case manager demographics**

**Variable**	**Case managers n = 178, n (%)**
Gender	
Female	161 (90.4)
Age (years)	
<40	45 (25.3)
40-49	41 (23.0)
50-59	71 (39.9)
>60	21 (11.8)
Training classification	
Nursing	75 (42.1)
Social work	36 (20.2)
Occupational therapy	4 (2.2)
Psychology	3 (1.7)
Community services	15 (8.4)
Disability services	5 (2.8)
Aged care/care coordination	15 (8.4)
Case management	15 (8.4)
Other	41 (23.0)

**Table 2:** **Service profiles**

**Variable**	**Service managers n = 120, n (%)**
State/territory	
NSW	25 (20.8)
NT	6 (5.0)
QLD	29 (24.2)
SA	8 (6.7)
TAS	12 (10.0)
VIC	36 (30.0)
WA	12 (10.0)
Service funding	
State funded	6 (8.2)
Private	3 (4.1)
Local government	6 (8.2)
Not for profit	52 (71.2)
Other	6 (8.3)
Service provides direct care to clients	
Yes	66 (90.4)
No	7 (9.6)
Service also provides residential aged care services	
Yes	48 (65.8)
No	25 (34.2)

### ACP systems and support

The service manager’s survey revealed that only 34% (n = 41) of services had written ACP policies and procedures in place and, although 64% (n = 77) reported that they offered ACP to clients, only 35% (n = 42) had ACP in the case manager job description.

Of the 36% (n = 43) of services which did not offer ACP to any clients the reasons for not offering ACP were: the organisation did not have a policy or procedure (69%), lack of ACP training for staff (52%), lack of skills with ACP (45%), the organisation had never identified ACP as a need (29%) and time and resource limitations (10%).

The majority of case managers were not satisfied with the level of support from their service to complete ACP with clients. Specifically, 65% (n = 116) were not satisfied with time allowed to undertake ACP, 60% (n = 107) with lack of support from senior staff to discuss the issues, 67% (n = 119) with lack of appropriate documentation for recording outcomes of discussions, 78% (n = 139) with lack of training to facilitate ACP discussions and 72% (n = 128) with absence of written information to give to services users and their family about ACP. In addition, only 36% (n = 64) of case managers believed they had sufficient time in their workload to complete ACP, only 27% (n = 48) believed that the majority of clients were interested in ACP, and only 15% (n = 27) of case managers believed ACP was done well within their service. Case managers were mostly (56%, n = 100) satisfied with support from peers to discuss ACP with clients.

### ACP knowledge and confidence

**Figure 1:**
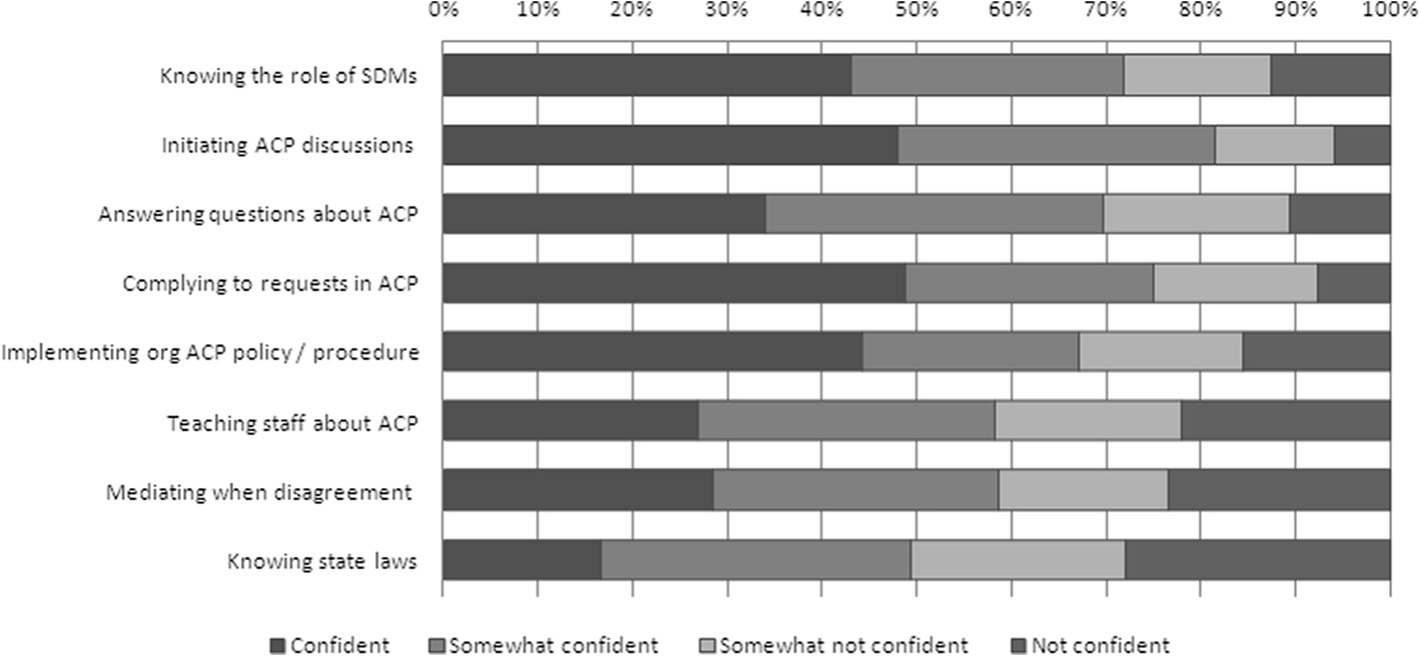
Case manager’s confidence levels with advance care planning processes.

Only 48% (n = 85) of all case managers had previously completed any ACP training and this training was only funded by the service 30% (n = 25) of the time. Despite this lack of ACP training, 66% of (n = 117) case managers felt comfortable discussing ACP with clients, however; only 32% (n = 57) believed they were skilled or very skilled to complete ACP discussions and only 30% (n = 53) of all case managers believed they were sufficiently trained to complete ACP discussions with clients. Less than 50% of case managers felt confident regarding their knowledge and skills on eight separate ACP domains (see Figure 1). In addition, no significant relationship was detected between having a nurse training background and confidence levels on the eight separate ACP domains.

### ACP discussions

In total, 36% (n = 43) of service managers reported that ACP is not offered to any clients, 22% (n = 27) to some clients (with no particular rationale), 17% (n = 21) to some clients who meet specific criteria only and only 24% (n = 29) offered ACP to all clients.

#### Examples of specific criteria/conditions that ACP is offered to clients as specified by service managers

- “Client’s request or expression of concerns/wishes [regarding] future health care and declining health”.
- “Client’s family may raise concerns”.
- “In conjunction with the Palliative care team if the client is also under the Palliative care team care”.
- “If and when it has been identified that a person my require ACP”.
- “If client does not have ACP in place”.
- “Client must be capable of making an informed decision. No dementia diagnosis”.
- “Clients who may be palliative and residing in their own homes”.
- “We have a brochure in all of the client’s home files but we raise it particularly when we can see there may be some issues for that person going forward, if they have not already completed their Advanced Care Plan”.

The case manager survey showed that, although 70% (n = 125) had initiated an ACP conversation in the past 12 months, 80% of the conversations did not progress to documentation of the client’s wishes. 47% (n = 84) scheduled further meetings with family and significant others, 19% (n = 34) referred the services user to an ACP service (within an existing health service/hospital), 45% (n = 80) made a referral to the client’s GP, 20% (n = 36) took no further action and 32% (n = 57) reported that the client refused to proceed further.

#### Description of ACP conversation outcomes as specified by case managers

- “Client to follow up with doctor and family”
- “Client only discussed her plans – put into action independently”
- “We would only send out information and Advance Care Planning documentation to the client but don’t usually help facilitate the process”
- “The next of Kin will take over and usually arrange for the client”
- “It’s noted if client has an advance care plan”
- “Documents are often completed and left with client for GP to sign”
- “Wishes recorded in documentation”

### Attitudes towards ACP for clients

Case managers were generally positive about ACP for clients; 75% (n = 134) believed that ACP was valuable and worthwhile for clients, 69% (n = 123) agreed that ACP is as important in HCPs as in RACFs and 74% (n = 132) believed clients are more comfortable discussing ACP and EOL care in their own home. Furthermore, 55% (n = 98) of case managers believe they have a role to play in providing ACP, 55% (n = 98) disagree that discussing death is a barrier to ACP and 57% (n = 101) felt confident discussing death and dying with their clients. Only 12% (n = 21) of case managers reported having had a negative experience with ACP.

## Discussion

This is the first published national survey to examine current ACP practice among Australian HCP case managers and service managers. This survey showed that the uptake of ACP in HCP settings is low, with case managers reporting they are under supported to undertake ACP discussions with clients and lack experience and training with ACP. These results are similar to published international research [15,17,19,22].

The participating HCP services were representative of Australian HCP services, and the gender and professional characteristics of our HCP case manager respondents were similar to the case manager population in the largest state of Australia in New South Wales [23], where the majority are female and from a nursing, allied health or aged/disability care background.

Consistent with international literature examining the state of ACP within HCPs [15-18], our survey revealed lack of systems and support to complete ACP, and low uptake with absence of a normative approach. For example, international studies show the majority of case managers have not received any ACP training within their organisation and, in turn, they report limited ACP knowledge and skills [15,17,19]. We also found that the majority of case managers had not completed any ACP training (52%) and that the majority of services lacked ACP policies or guidelines (66%) and did not include ACP in the case manager job description (65%). Furthermore, the majority of case managers were less than confident with their knowledge and skill in relation to aspects crucial to ACP implementation (such as knowledge of state laws and documenting patient wishes in an ACD).

A recent North American survey showed that case managers do not routinely initiate ACP with clients, despite the majority reporting past experience with ACP, holding the belief that most clients needed ACP and having high comfort levels in discussing the issues [22]. Similarly in our study, although 70% of case managers reported having had 1–12 discussions in the past 12 months, only 20% of those discussions progressed to documentation of treatment preferences. This was despite the majority of case managers (75%) believing ACP was valuable and worthwhile for clients and that ACP was part of their role (55%). Only 15% believed ACP was done well within their service.

The findings in this survey are consistent with the state of ACP within home care service providers [15-18] and with research across many other healthcare settings including where elderly patients with chronic disease do not have access to best-practice ACP [24], such as RACFs [20], general practice [25,26], and in acute and outpatient settings [13,27].

With the prevalence of HCPs increasing [10], coupled with the increased attention to conduct ACP outside of acute and RACF settings, there is a strong impetus to provide ACP in HCPs. Past evaluations of whole-systems–ACP-interventions have mostly occurred in the acute setting [4] or in RACFs [28,29], however no such interventions have been examined in the community setting, where clients are exposed to a more fragmented health system, with many health professionals or services [23]. Internationally, case managers have been shown to view their role as not to complete ACP documents, but to provide information and encourage consumers to talk with family and clinicians [19].

Although 70% of case managers in this study had initiated 1 or more ACP discussions in the last 12 months, only 20% resulted in documentation. What is the best way to increase the effectiveness? The two alternatives are to: 1. train the case managers to facilitate ACP, including completion of documents, or 2. to provide information and resources to guide them to refer clients externally.

To date, no studies have explicitly measured whether a ‘referral only’ model of ACP is effective or sustainable, or whether case managers are equally as effective at initiating and documenting ACP discussions themselves, when provided with the appropriate training and resources. Nursing, allied health workers and aged/disability care workers make up half of the case manager workforce [23] and past research shows these professionals are well suited to complete ACP when given the appropriate training [4,12]. Furthermore a recent Australian study has shown that education is effective at improving doctors’ confidence in undertaking ACP discussions, and was well received [26].

We believe a ‘referral only’ model could fit with the current practice of case managers in this sample who discussed ACP with their clients, and where the most frequent outcomes of ACP discussions were to schedule meetings with significant others (47%) and to refer to the client’s GP (45%). This model is supported by the open-ended responses from case managers indicating that most clients are currently referred elsewhere to complete ACP. One challenge is to identify the ACP resources that are required to provide training on ACP to the case managers who are, in most cases in Australia [23] and our survey, from a non-nursing background. There is no doubt, however, that resources to improve ACP knowledge and skills are required if we expect case managers to complete these types of discussions in their current role.

There are several limitations to the generalisability of these findings to HCP services across Australia. Firstly, as the survey relied on self reporting by the respondents regarding their practices and experiences with ACP, this may have resulted in over or underestimating prevalence of practices and in socially desirable responding. To counter this potential bias we ensured that the survey was anonymous and that questions were specific to HCP services and phrased to optimise objectivity. A second limitation is that the response rate was relatively low; this is a recognised limitation of the online survey format [30]. In order to ensure anonymity, we did not link the survey responses to the services provided in the contact list. We were not, therefore, able to determine the characteristics of services that did not respond to the survey. Although this potential limitation is a deliberate trade-off to ensure anonymity, the characteristics of services and the profiles of case managers were similar to past surveys suggesting that the characteristics of the services and staff that did not respond to our survey would be similar to Australian HCP services [11,23].

Given that the prevalence of care services for the elderly generally [31] and for home care service providers specifically [10] is expected to increase, the next step for research in this area is, firstly, to identify different possible models for delivering effective ACP to recipients of HCP services and then to test them in a controlled way. Such ACP models should aim to overcome barriers and facilitate ACP at the case manager, client and caregiver and system levels. In accordance with the facilitators and barriers identified in this study we propose that the ‘referral only’ model and ‘case manager facilitator’ model should be further developed and tested in a Randomised Controlled Trial. For example, this study identifies that case managers believe they have a role in ACP and that it is worthwhile for their clients; however, they do not currently have the systems and support at the organisational level to facilitate ACP discussions. It is currently unclear which model would improve the uptake of ACP among HCP clients.

Furthermore, to best evaluate future ACP interventions we recommend studies measure outcomes that relate to the known benefits of ACP generally and also the foreseeable benefits of ACP for HCP clients specifically. Such outcome measures should include: the likelihood that doctors and family members know and comply with the patients’ wishes [3-6] and patient and family satisfaction with care, as well as reduced stress, anxiety and depression in surviving relatives [4-6,9]. Therefore, we will not only be able to determine whether the proposed model increases the uptake of ACP among HCP clients, but also whether it improves end-of-life outcomes for HCP clients, including whether their wishes are known and respected by the family and doctors.

## Conclusions

This is the first national survey examining the current state of ACP within HCPs. The findings are consistent with past international research, which shows that there is a low uptake and lack of normative approach of ACP in HCP services. This is in spite of the fact that case managers view ACP as valuable and worthwhile for their clients and that the majority view initiating ACP as part of their role. HCP case managers require greater support within their organisations, including greater access to ACP training and policies and guidelines.

## Endnote

^1^The response rates are made on the assumption that each service followed the study protocol by passing on the request to complete the online survey to two case managers and one service manager.

## Additional file

Additional file 1:
**HCP_surveys.**

## Abbreviations

ACP: Advance care planning
ACD: Advance Care Directive
RACF: Residential aged care facility
HCP: Home Care Packages
