# Supplementary material for: Current advance care planning practice in the Australian community: an online survey of home care package case managers and service managers
Source: BMC Palliat Care. 2015 Apr 23;14:15. doi: 10.1186/s12904-015-0018-y (PMC4416336; doi:10.1186/s12904-015-0018-y)
Supplement: Additional file 1: — HCP_surveys. [file 12904_2015_18_MOESM1_ESM.pdf]

1. Case Manager Survey

Demographics

1. Gender
2. How old are you?
3. What is your professional training classification? (social work / psychology / occupational therapy / nursing / other)
4. What is the position held within the organisation of person completing this survey:
5. What is your service provider type?
   - State funded
   - Private
   - Local government
   - Not for profit (charitable, community based, religious affiliation)
   - Other (please specify)
6. Does your organisation also provide Residential Aged Care services? (Y/N)
7. Does your service provide direct care to service users? (Y/N)
8. Please estimate, as accurately as possible, the total amount of your individual current service user caseload within the last week.
9. In which state/territory is your Community Aged Care service (Tick all that apply)? (ACT, NSW, NT, QLD, SA, TAS, VIC, WA)
10. Where is the location of your service (Tick all that apply)?
    - Within a major city
    - Inner regional
    - Outer regional
    - Remote
    - Other (please specify)

ACP policy and procedures

An Advance Care Plan is a written or verbal record of a person's choices about their future medical care. An Advance Care Plan is only used when the person is no longer able to make or communicate their own decisions about their medical treatment. It works as a guide to the substitute decision makers and doctors who will make medical treatment decisions in the future if the person becomes incapable of making or communicating such decisions.

1. Does your service have written policies and procedures concerning Advance Care Planning? (Y/N/Don't know)
2. If yes, have you read the policies and procedures concerning ACP in your service? (Y/N)
3. Have you undertaken training about Advance Care Planning?
   - Yes, in the last 2 years
   - Yes, more than 2 years ago
   - No
   - Don't know
4. Was any of the ACP training funded by your Commonwealth Packaged Care service?
   - All training provided by Commonwealth Packaged Care service
   - Some training provided by Commonwealth Packaged Care service
   - None of the training was provided by Commonwealth Packaged Care service
5. Where Advance Care Planning training was funded by your organisation, how was it delivered (tick all that apply)?
   - In-service
   - External workshop
   - Online training
   - Other (please specify)
6. Where Advance Care Planning training was NOT funded by your organisation, how was it delivered (tick all that apply)?
   - In-service
   - External workshop
   - Online training
   - Other (please specify)

Experiences with ACP

1. In your experience, who usually initiates discussions with client/families about advance care planning, deterioration of health and / or end of life care (Tick all that apply)?
   - Not applicable – ACP discussions are not completed with clients
   - Aged Care Assessment Team / Aged Care Assessment Service
   - I initiate discussions
   - Other Commonwealth Packaged Care staff
   - Service user
   - Family or friends of service user
   - GP / specialist
   - Community palliative care service
   - Hospital staff when patients are admitted
   - Other (please specify)

2. How well do you think ACP is currently done in your service? (very poorly, poorly, somewhat poorly, somewhat well, well, very well)
3. Rate your level of support with the way your service has supported you to discuss ACP with service users and their families (very unsatisfactory, unsatisfactory, somewhat unsatisfactory, somewhat satisfactory, satisfactory, very satisfactory):
    - Time allowed to undertake ACP
    - Support from senior staff / service owners to discuss the issues
    - Appropriate documentation for recording outcomes of discussions
    - Training to facilitate these discussions
    - Support from peers
    - Written information to give to service users and families to support ACP
4. In the past year, how many of your service users have you had discussions about ACP / deterioration of their health / end-of-life care?
5. When ACP discussions have been initiated what has been the outcome of these discussions? Tick all that apply.
    - Further meetings with significant others
    - A referral to an ACP service
    - A referral to a GP
    - The case manager facilitates ACP document completion
    - No further action
    - The client refused to proceed further
    - Other (please specify)
6. How confident are you, or would you be about undertaking the following activities / roles in ACP? (very confident, confident, somewhat confident, somewhat not confident, not confident, not at all confident)
    - Knowing the role of substitute decision makers
    - Initiating ACP discussions with service users
    - Answering questions about ACP
    - Complying to requests in the service users' ACP
    - Implementing ACP policy / procedures
    - Teaching staff about ACP
    - Mediating when there is disagreement between service users and family members regarding EOL decisions
    - Knowing state laws regarding ACP
7. How comfortable are you, or would you be, discussing ACP / deterioration of their health / end-of-life care? (very comfortable, comfortable, a little comfortable, uncomfortable, very uncomfortable)

8. How skilled do you feel, or would you feel, discussing ACP / deterioration of their health / end-of-life care? (very skilled, skilled, a little skilled, skilled, very skilled)

Professional attitudes, values, and beliefs about ACP

1. Please rate your level of agreement for the following statements about ACP in your Commonwealth Packaged Care service (strongly agree, agree, slightly agree, slightly disagree, disagree, strongly disagree):
   - The majority of service users are interested in ACP
   - I have sufficient time in my workload to completed ACP discussions with service users
   - I have no role in ACP
   - ACP is a worthwhile and valuable activity for my service users
   - I have not been sufficiently trained to discuss death dying with service users
   - For me, discussing death is a barrier to ACP discussions
   - I do not feel confident discussing death and dying with service users
   - I am very experienced at ACP discussions
   - ACP is not as important in the Community Aged Care setting as it is in Nursing Homes
   - I have had a negative experience with ACP
   - The service user's family play an important role in the initiation of ACP discussions
   - In my opinion, the service user is more comfortable discussing end-of-life care in their own home setting
   - It should be the General Practitioners role to complete ACP with service users

<u>End of Survey</u>

2. <u>Service Manager survey</u>

Demographics

1. What is the position held within the organisation of person completing this survey:
2. What is your service provider type?
   - State funded
   - Private
   - Local government

- Not for profit (charitable, community based, religious affiliation)
- Other (please specify)

3. Does your organisation also provide Residential Aged Care services? (Y/N)
4. Does your service provide direct care to service users? (Y/N)
5. Within your organisation, how many services currently provide Commonwealth Packaged Care services?
6. Please specify the total number of Commonwealth packages in your organisation within the last week.
7. In which state/territory are your Community Aged Care service (Tick all that apply)? (ACT, NSW, NT, QLD, SA, TAS, VIC, WA)
8. In which location(s) are your organisation's Community Aged Care services (Tick all that apply)?
   - Within a major city
   - Inner regional
   - Outer regional
   - Remote
   - Other (please specify)

ACP policy and procedures

An Advance Care Plan is a written or verbal record of a person' s choices about their future medical care. An Advance Care Plan is only used when the person is no longer able to make or communicate their own decisions about their medical treatment. It works as a guide to the substitute decision makers and doctors who will make medical treatment decisions in the future if the person becomes incapable of making or communicating such decisions.

1. Does your organisation currently formally record service users' wishes about the care they would like to receive in case they are no longer able to make decisions about their health for themselves (i.e. Advance Care Planning)? (Y/N)
2. Does your organisation have written policies and procedures concerning Advance Care Planning? (Y/N)
3. Is ACP the role of case managers? (Y/N)
4. Is ACP part of the case manager's job description? (Y/N)

ACP practice

1. Currently, to what proportion of service users in your organisation is Advance Care Planning offered?
   - ACP is offered to all service users

- ACP is offered to some service users – but with no particular rationale
- ACP is offered to some service users who meet specific criteria (please specify below)
- Not applicable – ACP is not offered to service users

2. Please specify any reasons Advance Care Planning is not offered to service users (Tick all that apply):
    - The organisation doesn't have a policy or procedure
    - The organisation has never identified this as a need
    - Time and resource limitations
    - Lack of skills with ACP
    - Lack of ACP training for staff
    - Lack of confidence with ACP
    - Service users and families do not want to complete ACP
    - Other (please specify)

3. Are service user's completed Advance Care Planning documents transferred to other health services / providers (such as GP or local hospital or residential aged care facility or respite facility)? (Y/N)
